# Focus on Nivolumab in NSCLC

**DOI:** 10.3389/fmed.2016.00067

**Published:** 2016-12-13

**Authors:** Diego L. Cortinovis, Stefania Canova, Marida Abbate, Francesca Colonese, Paolo Bidoli

**Affiliations:** ^1^SC Oncologia Medica, Ospedale San Gerardo, Monza, Italy

**Keywords:** nivolumab, immunotherapy, NSCLC, PD-1, PDL1, checkpoint inhibitors

## Abstract

Immunotherapy is changing the treatment of non-small cell lung cancer (NSCLC). The PD-1 inhibitor nivolumab has demonstrated meaningful results in terms of efficacy with a good safety profile. The novel approach to treating NSCLC using immunotherapy still has unsolved questions and challenging issues. The main doubts regarding the optimal selection of the patient are the role of this drug in first line of treatment, the individualization of the correct methodology of radiologic assessment and efficacy analysis, the best management of immune-mediated adverse events, and how to overcome the immunoresistance. The aim of this review is to analyze literature data on nivolumab in lung cancer with a focus on critical aspects related to the drug in terms of safety, the use in clinical practice, and possible placement in the treatment algorithm.

## Rationale for Immune Checkpoint Inhibitors

Several clinical observations foresaw the promising results arising from the employment of immunotherapy in lung cancer. Indeed, the lungs are involved in many autoimmune disorders. In addition to hyperplasia of fibroblasts, diminished collagen breakdown and production of autoantibodies, the pathophysiology of pulmonary disease includes activation of T cells, B cells, and alveolar macrophages. Activated T cells produce cytokines, such as interleukin-4 and interleukin-10, which enhance fibroblasts proliferation. Furthermore, activated T cells produce an altered form of interferon gamma (IFNγ) with a reduced skill to inhibit fibroblasts proliferation (1). Moreover, spontaneous tumor regressions, not only in cutaneous melanoma (2) but also in lung cancer (3), have been described and confirm the involvement of immune system in cancer control.

At the beginning of the twentieth century, Paul Ehrlich first proposed the idea that transformed cells can elicit immune system to repress them (4). The discovery of rejection of transplanted tumors in mice and the existence of tumor-associated antigens (5) led then Burnet to propose the hypothesis of cancer immune surveillance (6) with the assumption that “tumour cells provoke an effective immunological reaction with regression of the tumor and no clinical hint of its existence” (6). However, immune surveillance is not sufficient to explain the occurrence and growth of cancer in immunocompetent individuals. Indeed, tumors acquire ability to resist to host’s immune system. The term “cancer immunoediting” has been proposed to explain this complex interaction between cancer and host and includes three phases: elimination, equilibrium, and escape. During elimination phase, tumor growth induces the release of inflammatory signals that activate cells of the innate immune system. These are natural killer (NK), NK T cells, γδ T cells, macrophages, and dendritic cells (DCs). They produce IFNγ, which has antiproliferative and apoptotic effect, and induce chemokines such as CXCL10, CXCL9, and CXCL11. These chemokines block angiogenesis and recruit more NK and macrophages that promote the maturation of DCs. DCs capture necrotic tumor cells, migrate to lymph nodes, and present tumor antigens (TAs) to naïve CD4^+^ T cells leading to their differentiation in effector CD4^+^ T cells, development of TA-specific CD8^+^ T cells, and their expansion. Finally, TA-specific T cells can home to tumor site and eliminate tumor cells. Some tumor cells that withstand the elimination phase enter the equilibrium process. During this phase, activated T cells and IFNγ manage to limit tumor growth without removing it. Nevertheless, tumor cells with reduced immunogenicity for low levels of TAs survive and become resistant to immune system. They enter the escape phase and expand in an uncontrolled way (7). To become effector T cells, naïve T cells must recognize their specific TAs and interact with DCs through major histocompatibility complex. This interaction involves both costimulatory and coinhibitory signals. In normal tissues, there is a balance between these signals. By contrast, inhibitory receptors and ligands are overexpressed on tumor cells and in tumor microenvironment. For example, high proportions of CD4^+^CD25^+^ T cells are present in the tumor-infiltrating lymphocytes (TILs) of patients with non-small cell lung cancer (NSCLC) (8). These T cells show high expression of CTLA-4 on their surface and inhibit the activation of T cells (9).

Immune checkpoints, such as CTLA4 and PD-1, are crucial to maintain the balance between costimulatory and inhibitory signals limiting excessive immune response against self-antigens. Thus, they are potential targets for cancer therapies.

CTLA4 is expressed on CD8^+^ T cells, CD4^+^ T cells, and on regulatory T cells (T_reg_) and is involved in early stages of T cell activation. Its ligands are CD80 (B7.1) and CD86 (B7.2) expressed on antigen-presenting cells (APCs) like DCs (10). CD28 is a costimulatory receptor also expressed on T cells, which binds to CD80 and CD86 with consequent activation of T cells. CTLA4 interacts with CD80 and CD86 with higher affinity than CD28 does and inhibits CD4^+^ T cell activation (11).

Even though CTLA4 is expressed by activated CD8^+^ effector T cells, the major physiological role of CTLA4 seems to be through distinct effects on the two major subsets of CD4^+^ T cells: downmodulation of helper T cell activity and enhancement of T_reg_ activity. The latter is crucial for the maintenance of self-tolerance (12).

PD-1, as CTLA4, is expressed on T cells, but contrary to CTLA4, it is involved in the late phases of immune reactions and mostly within the tumor microenvironment. Its ligands are PD-L1 (B7-H1) and PD-L2 (B7-DC) that are expressed on APCs and tumor cells. The interaction of PD-1 with its ligands results in reduced effector T cell proliferation, exhaustion of T cell activity, and enhancement of T_reg_ proliferation (13). Tumors are able to escape immune control because of upregulation of PD-1 on their surface. Indeed, PD-L1 is expressed in about 50% of NSCLC, mostly in squamous subtypes at advanced stage, and seems to correlate with poor prognosis (14, 15).

Two mechanisms of PD-1 ligands upregulation are present, known as innate immune resistance and adaptive immune resistance. The first refers to the constitutive expression of PD-L1 through involvement of oncogenic signaling pathways, such as AKT and STAT3, as in ALK-positive lung cancer (16, 17). In adaptive immune resistance, PD-1 ligands are overexpressed on tumor cells in response to cytokines, in particular IFNγ (18). The adaptive immune resistance is probably involved in most NSCLC without an oncogenic driver. Indeed, higher neoantigen burden seems associated with clinical benefit of PD-1 blockade (19).

Due to strong rationale and promising preclinical data, monoclonal antibodies anti-CTLA4 and anti-PD-1/PD-L1 have been extensively studied in advanced NSCLC. The therapeutic interference of immune synapse was a strategy adopted in preclinical model from 2010, and nivolumab was the “first in class” MoAb to be employed in clinical trials in advanced NSCLC immediately the unripe experience of Ab anti-CTLA4.

## Nivolumab Development in Clinical Practice: State of the Art

Nivolumab was evaluated in the Phase Ib dose-escalation trial Checkmate 003 (20) (Table 1) in 129 heavily pretreated NSCLC patients. It was administered at 1, 3, and 10 mg/kg i.v. every 2 weeks for up to 96 weeks. Median OS for 3 mg/kg cohort was longer than mOS for 1 and 10 mg/kg (14.9 vs. 9.2 months). Median progression-free survival (mPFS) was 2.3 months, median duration of response was 17.0 months, and the overall response rate (ORR) was 17%, similar for squamous and non-squamous NSCLC. Eighteen patients discontinued the study without progression and 50% of these continued to respond 9 months after the last dose. The dose of 3 mg/kg every 2 weeks of nivolumab was determined as the dose to be employed in further trials.

**Table 1:** **Major clinical trials of nivolumab in lung cancer**.

Trial	No. patients	Phase	Histology	Setting	Treatment	Outcome	Safety	Notes
*CheckMate 003* (20)	129	Phase I	Non-small cell lung cancer (NSCLC)	Pretreated	Nivolumab dose escalation	OS 3 mg/kg 14.9 months vs. mOS 1 and 10 mg/kg 9.2 months	3 treatment-related deaths (associated with pneumonitis)	

*CheckMate 063* (21)	117	Phase II	Squamous NSCLC	Pretreated	Nivolumab 3 mg/kg	OS 8.2 months 1-year OS 41%	17% of the pts reported Grade 3 or 4 treatment-related AEs. Two treatment-associated deaths (pneumonia and ischemic stroke)	PD-L1 cutoff of 5%; nivolumab demonstrated activity irrespective of PD-L1 expression

*CheckMate 017* (22)	272	Phase III	Squamous NSCLC	Pretreated	Nivolumab vs. docetaxel	OS 9.3 vs. 6.0 months	Grade 3 or 4 treatment related were reported in 7% of the pts in the nivolumab arm vs. 55% in the docetaxel arm	Nivolumab demonstrated activity irrespective of PD-L1 expression

*CheckMate 057* (23)	582	Phase III	Non-squamous NSCLC	Pretreated	Nivolumab vs. docetaxel	OS 12.2 vs. 9.4 months	Grade 3 or 4 treatment-related AEs were reported in 10% of the pts in the nivolumab arm vs. 54% in the docetaxel arm	PD-L1 cutoff ≥1, ≥5, and ≥10%; relevant predictive association between OS, median progression-free survival, overall response rate (ORR), and PD-L1 expression

*CheckMate 012* (24)	52	Phase I	NSCLC	I line	Nivolumab 3 mg/kg	OS 19.4 months 12-month OS 73%	19% of pts reported Grades 3–4 treatment-related AEs; 12% discontinued because of a treatment-related AE	PD-L1 cutoff ≥1 and <1%, ≥5 and <5%; clinical activity regardless of PD-L1 expression, but higher ORR for greater PD-L1 expression. Not clear correlation between PFS, OS, and PD-L1 expression

*CheckMate 012* (25)	56	Phase I	NSCLC	I line	Nivolumab + platinum-based doublet chemotherapy (PT-DC)	OS PT-DC + Nivo 10 mg/kg from 11.6 to 19.2 months; plus Nivo 5 mg/kg not reached	45% of pts reported Grade 3 or 4 treatment-related AEs. 21% of pts discontinued because of a treatment-related AEs	Nivolumab demonstrated activity irrespective of PD-L1 expression

*CheckMate 032* (26)	216	Phase I/II	Small cell lung cancer	Pretreated	Nivolumab or sequentially cohorts nivolumab + ipilimumab	OS Nivo 4.4 months; OS Nivo + IPI 6–7.7 months; 1-year OS 33 and 35–43%	Grade 3 or 4 treatment-related AEs events occurred in 13% of pts in the nivolumab 3 mg/kg cohort, 30% in the nivolumab 1 mg/kg + ipilimumab 3 mg/kg, and 19% in the nivolumab 3 mg/kg + ipilimumab 1 mg/kg. Two pts who received nivolumab 1 mg/kg + ipilimumab 3 mg/kg died from treatment-related AEs (myasthenia gravis and renal failure); 1 who received nivolumab 3 mg/kg + ipilimumab 1 mg/kg died from treatment-related pneumonitis	No correlation between PD-L1 expression and response

CheckMate 063 (21) (Table 1), a Phase II, single-arm trial, evaluated nivolumab activity in 117 pretreated advanced squamous NSCLC patients. ORR was the primary endpoint. About 14.6% (17/117) of patients obtained a response, 26% (30/117) had stable disease (SD). Response was achieved in a median time of 3.3 months, and the majority of responses were ongoing at the time of the report. Patients with SD had a duration of response of 6 months. Nivolumab demonstrated activity irrespective of PD-L1 expression, using a cutoff of 5%. PD-L1 was assessed in 76 patients, 33% (25/76) had PD-L1 expression and among them 6 patients had a partial response, whereas 7 patients of 51 with PD-L1-negative obtained a response.

After these promising results, nivolumab was compared with chemotherapy in two randomized Phase III trials in second line in advanced squamous and non-squamous NSCLC.

CheckMate 017 (22) (Table 1), a randomized open-label Phase III trial, employed nivolumab or docetaxel in advanced squamous (SCC) NSCLC after progression to first-line chemotherapy. OS was the primary endpoint, and it was significantly longer in the nivolumab arm compared to docetaxel (9.3 vs. 6.0 months). Nivolumab decreased the risk of death of 41% (hazard ratio 0.59; 95% CI, 0.44–0.79; *P* < 0.001). In the experimental arm, ORR (20 vs. 9%) and PFS (3.5 vs. 2.8 months; hazard ratio for death or disease progression, 0.62; 95% CI 0.47–0.81; *P* < 0.001) were also increased.

There was no correlation between PD-L1 expression and nivolumab activity (PD-L1 analysis was performed retrospectively).

Nivolumab was also compared to docetaxel in the CheckMate 057 (23) (Table 1), a randomized Phase III trial in non-squamous advanced NSCLC after platinum-based doublet chemotherapy (PT-DC). OS was the primary endpoint, and as previously seen in SCC, it was improved for nivolumab-treated patients (12.2 vs. 9.4 months, hazard ratio for death, 0.73; 96% CI, 0.59–0.89; *P* = 0.002). OS rate at 1 year and 18 months was longer for the experimental arm (51 and 39% vs. 39 and 23%) in addition, there was an advantage also for ORR (19 vs. 12%) with a longer duration of response and a median time to response of 2.1 vs. 2.6 months. Immunotherapy was not superior to chemotherapy in terms of mPFS (2.3 and 4.2 months). PD-L1 expression was assessed retrospectively on archival or recent tumor tissue. PD-L1 cutoff was ≥1, ≥5, and ≥10%. It was observed a relevant predictive association among OS, mPFS, ORR, and PD-L1 expression. Subgroup analysis revealed that patients who received third line of chemotherapy, the presence of central nervous system metastases, EGFR mutation, and patients who lived in South America, Asia, and Australia obtained more benefits from chemotherapy. Kaplan–Meyer curves of OS and PFS revealed a chemotherapy early advantage, however, later curves crossed showing a nivolumab advantage. This unexpected finding may be explained by an initial benefit from chemotherapy in patients who do not expressed PD-L1 but presented EGFR mutations. In fact, in this setting, the experimental drug provided less advantage respect to chemotherapy By contrast, in CheckMate 017 trial, Kaplan–Meyer curves had an early separation, particularly for OS. It can be related to nivolumab benefit in overall squamous NSCLC population.

CheckMate 012 trial (24, 25) (Table 1) was conducted in I line in advanced NSCLC. It is a Phase I multicohort study that evaluated the safety and efficacy of nivolumab monotherapy or combined to PT-DC. Pretreatment tissue was used only for biomarker evaluation and not for patients’ selection. In monotherapy, nivolumab was administered to 52 patients. ORR was 23%, 27% of patients had SD with a disease control rate of 50%. mOS in overall population was of 19.4 months (16.8 months in squamous histology and NR in non-squamous), 12-month OS rate in overall population was 73% (76% in squamous histology and 72% in non-squamous), and 18-month OS rate in overall population was 57% (42% in squamous histology and 63% in non-squamous). In overall population, mPFS was 3.6 months and 24-week PFS was 41%.

Clinical activity was observed regardless of PD-L1 expression, and higher ORR was related to greater PD-L1 expression. The correlation between PFS, OS, and PD-L1 expression is not clear. Smoking history seems to be associated with higher activity of nivolumab. In the combination arm, nivolumab was administered to 56 patients for four cycles every 3 weeks at 10 mg/kg + cisplatin–gemcitabine in squamous histology, plus cisplatin–pemetrexed in non-squamous histology or at dose of 5 or 10 mg/kg + carboplatin–paclitaxel in all histologies. After the planned chemotherapy cycles, patients received nivolumab alone. Nivolumab dose of 5 mg/kg was emended when trial was ongoing. mPFS ranged from 4.8 to 7.1 months, 24-week PFS rate from 38 to 71%. Range of mOS of PT-DC + nivolumab at 10 mg/mg was from 11.6 to 19.2 months, but it was not reached for nivolumab at 5 mg/kg + carboplatin–paclitaxel. ORR was 48% for patients with PD-L1 expression >1 and 43% if PD-L1 was <1%. Nivolumab activity also occurred if PD-L1 was absent or low expressed, whereas smoking history was related to higher clinical activity.

Small cell lung cancer (SCLC) is strongly related to tobacco use, and as a result, it is characterized by high mutational burden. Response to second-line chemotherapy is around 9–23% depending on platinum sensitivity.

CheckMate 032 (26) (Table 1) is a muticentre, Phase I/II open-label trial. Patients affected by limited or extended SCLC, after at least platinum-based chemotherapy, received: nivolumab 3 mg/kg every 2 weeks, nivolumab + ipilimumab every 3 weeks for four cycles (1 + 1, 1 + 3, and 3 + 1 mg/kg), then nivolumab 3 mg/kg every 2 weeks. Patients were enrolled sequentially in the four cohorts. The cohort nivolumab 1 mg/kg + ipilimumab 1 mg/kg is the smaller with only 3 patients of 216 overall patients. At interim analysis, ORR was 10% for nivolumab, 23% for nivolumab 1 mg/kg + ipilimumab 3 mg/kg, and 19% for nivolumab 3 mg/kg + ipilimumab 1 mg/kg. mOS was 4.4 months for nivolumab, 7.7 months for nivolumab 1 mg/kg + ipilimumab 3 mg/kg, and 6.0 months for nivolumab 3 mg/kg + ipilimumab 1 mg/kg. One-year overall survival was 33, 43, and 35%. mPFS was 1.4, 2.6, and 1.4 months. Most frequent Grade 3 or 4 AEs were diarrhea and increase of lipase occurring in 4, 30, and 15%. PD-L1 was evaluated retrospectively on archival or fresh tissue collected. PD-L1 expression in SCLC was lower compared to NSCLC, and there was no correlation found between PD-L1 and response.

This trial evidenced similar responses between platinum-resistance and platinum-sensitive patients. The reason is probably due to the mechanism of action of immune checkpoint that is completely different from chemotherapy (i.e., topotecan), and it works better in presence of high mutational burden. No differences were found between patients pretreated with one or more line of chemotherapy. Unfortunately, the absence of randomization does not allow to a comparison between the different arms. Nivolumab achieves rapid and durable responses. The majority of nivolumab studies are limited by the evaluation of PD-L1 expression that can change over time, so tissue collection deriving from archival or recent biopsy does not offer a PD-L1 real status even if this point remains a major concern to debate.

Trials of immune checkpoint inhibitors used different test to establish PD-L1 expression, so there is no unique test for PD-L1 evaluation and a comparison among PD-1/PD-L1 inhibitors is not possible. For this reason, the Blueprint development group has proposed a way to compare different diagnostic assays for future clinical practice that requires validation.

Interesting future development of nivolumab (Table 2) in lung cancer are as adjuvant therapy (NCT02595944), after chemo-radiotherapy (NCT02768558), in association with RT in case of intracranial metastasis (NCT 02696993), as maintenance treatment (NCT02538666; NCT02713867), and in combination with ipilimumab/chemotherapy/TKIs (NCT02477826, NCT02785952, NCT02659059, NCT02154490, NCT02041533, NCT02613507, NCT02481830, and NCT02864251).

**Table 2:** **Selected future development of nivolumab in lung cancer**.

Trial	Phase	Histology	Setting	Treatment	Status	Association
CheckMate 227 NCT02477826	Phase III	Non-small cell lung cancer (NSCLC)	I line	Nivo, NIvo + IPI, Nivo + platinum-based doublet chemotherapy (PT-DC), PT-DC	Recruiting	CT and Immunotherapy

ANVIL NCT02595944	Phase III	NSCLC	IB–IIIA adjuvant	Nivo	Recruiting	Immunotherapy

Lung-MAP NCT02785952	Phase III	Squamous NSCLC	II line	Nivo, Nivo + IPI	Recruiting	Immunotherapy

CheckMate 451 NCT02538666	Phase III	ED-small cell lung cancer (SCLC)	Maintenance after I line CT	Nivo + Placebo, Nivo + Ipilimumab	Recruiting	Immunotherapy

CheckMate-026 NCT02041533	Phase III	NSCLC PD-L1^+^	I line	Nivo, investigator’s choice CT	Active, not recruiting	CT and Immunotherapy

Cisplatin and etoposide + RT followed by Nivo/placebo for locally advanced NSCLC NCT02768558	Phase III	NSCLC	Unresectable, medically inoperable disease, or patients who refuse resection stage IIIA or stage IIIB disease	Thoracic RT, cisplatin, etoposide ± Nivo	Not yet recruiting	RT, CT, and Immunotherapy

CheckMate 078 NCT02613507	Phase III	NSCLC	II line, after platinum-based CT	Nivo, docetaxel	Recruiting	CT and Immunotherapy

Phase I/II trial of nivolumab with radiation or nivolumab and ipilimumab with radiation for the treatment of intracranial metastases from NSCLC NCT02696993	Phase I/II	NSCLC	Stage IV metastatic disease with intracranial disease	Nivo + IPI + WBRT, Nivo + IPI + SRS	Not yet recruiting	RT and Immunotherapy

CheckMate 331 NCT02481830	Phase III	SCLC	II line, after platinum-based CT	Nivolumab, topotecan, amrubicin	Not yet recruiting	CT and Immunotherapy

CheckMate 384 NCT02713867	Phase III	NSCLC	Nivo 240 mg every 2 W vs. Nivo 480 mg every 4 W after up to 12 months of Nivo at 3 mg/kg or 240 mg every 2 W	Nivo 240 mg every 2 W vs. nivolumab 480 mg	Recruiting	Immunotherapy

CheckMate 568 NCT02659059	Phase II	NSCLC	I line	Nivo + IPI	Recruiting	Immunotherapy

Lung-MAP NCT02154490	Phase II/III	Squamous NSCLC	II line	Docetaxel, durvalumab, erlotinib, hydrochloride, FGFR, AZD4547, IPI, laboratory biomarker analysis, Nivo, palbociclib, rilotumumab, taselisib	Recruiting	Immunotherapy, CT, and target therapy

CheckMate 722 NCT02864251	Phase III	NSCLC EGFR mut, T790M	After 1 line EGFR TKI therapy	Nivo + IPO vs. Nivo + PEM + CDDP/CBDCA	Not yet recruiting	Immunotherapy, CT, and target therapy

*CT, chemotherapy; Nivo, nivolumab; IPI, ipilimumab; PEM, pemetrexed; W, week*.

## Nivolumab – Safety Profile

As mentioned earlier, nivolumab demonstrated an improvement over current available therapies with a risk profile acceptable relative to the clinical benefit offered.

### Phase I

In Phase I study of nivolumab, treatment-related select adverse events of any grade were observed in 41% of 129 patients with NSCLC, and the most common included skin, gastrointestinal, and pulmonary events (16, 12, and 7%, respectively). Grades 3–4 treatment-related adverse events occurred in 14% of cases, with fatigue (3.1%) and pneumonitis (2.3%) being the most common. There were three treatment-related deaths associated with pneumonitis. No clear relationships between the occurrence of pneumonitis and dose level or treatment duration were noted (20).

### Phase II

In the non-comparative Phase II trial (ONO-4536-06) conducted in Japanese population (currently not published), any grade drug-related adverse events were reported in 68% of patients. Decrease appetite, malaise, pyrexia, and rash were the most frequent toxicities. Grade 3/4 toxicities were experienced in 5.7%. Regarding the immune-related adverse events, the most common was skin rash (reported in 28% of patients), followed by endocrine (11.4%), pulmonary, gastrointestinal, infusion reactions (each occurring at 5.7%), and renal (2.9) toxicity. No Grade 3/4 toxicities occurred (27).

### CheckMate 063: SCC

In the Phase II, single-arm study CA209063 (CM063), any grade treatment-related adverse events were reported in 74% of patients and included fatigue (33%), decreased appetite (19%), nausea (15%), asthenia (12%), rash (11%), and diarrhea (10%). Grades 3–4 treatment-related adverse events were observed in 17% of subjects, with fatigue (4%), pneumonitis (3%), and diarrhea (3%) being the most frequent. Treatment-related adverse events led to discontinuation of the drug in 12% of patients. Immune-mediated adverse reactions, defined as cases requiring use of systemic corticosteroids with no clear alternative cause were immune-mediated pneumonitis (6.0%), hypothyroidism (4.3%), hyperthyroidism (1.7%), motor dysfunction (1.7%), rash (1.7%), adrenal insufficiency (0.9%), vasculitis (0.9%), colitis (0.9%), and renal dysfunction (0.9%). These immunological side effects were treated with administration of high-dose corticosteroids followed by a taper and interruption of nivolumab therapy. Of note, no patients were rechallenged with nivolumab following corticosteroid taper. Finally, two treatment-associated deaths (one due to pneumonia and one due to stroke) occurred (21).

### CheckMate 017

In the Phase III open-label randomized trial CheckMate 017 comparing nivolumab vs. docetaxel in SCC NSCLC, the incidence of adverse events was 58% in the nivolumab group vs. 86% in the docetaxel arm. The most frequent adverse events in patients treated with nivolumab were fatigue (16%), reduced appetite (11%), and asthenia (10%), whereas in patients treated with docetaxel, neutropenia (33%), fatigue (33%), alopecia (22%), and nausea (23%) were commonly observed. In the overall study population, treatment-related Grade 3/4 adverse events were more common with docetaxel (55%) with a high number of hematologic toxic events and infections. On the contrary, only 6.9% of patients in the nivolumab arm reported Grade 3/4 treatment-related adverse events, and they were commonly represented by fatigue, decreased appetite, and leukopenia. Overall, 3.1% of patients in the nivolumab arm discontinued treatment due to an AE compared with 10.1% for docetaxel. The most frequently reported (≥3% of patients) selected treatment-related AEs of any grade were hypothyroidism (4 vs. 0%), diarrhea (8 vs. 20%), and pneumonitis (5 vs. 0%) for nivolumab and docetaxel, respectively. Discontinuation due to toxicity issues occurred in 10% of patients on docetaxel, mostly due to peripheral neuropathy, while only 3% interrupted nivolumab mainly for pneumonitis. Finally, no treatment-related deaths were reported for patients treated with nivolumab, whereas three deaths occurred (one death each from interstitial lung disease, pulmonary hemorrhage, and sepsis) in docetaxel arm (22).

Data regarding longer follow-up of the study showed no unpredicted adverse events with nivolumab and a good safety profile compared to docetaxel (28).

### CheckMate 057: nsq NSCLC

In the Phase III CheckMate 057 having similar characteristics in terms of design, endpoints, drugs, and schedules of treatment of CheckMate 017, but with a larger samples (in the CheckMate 057:292 and 290 patients in the nivolumab and docetaxel arm, respectively, in the CheckMate 017:135 patients in the nivolumab arm and 137 in the docetaxel arm), the safety profile was in line with the previous reports. More in details, safety analysis demonstrated that AEs of any grade occurred in 69% of patients receiving nivolumab and 88% of patients receiving docetaxel. Among them, the most frequent were fatigue, nausea, decreased appetite, and asthenia in the nivolumab group, whereas neutropenia, fatigue, nausea, alopecia, diarrhea, and anemia were the most common in the docetaxel group. Treatment-related Grade 3/4 adverse events were reported by 10% of the patients treated with nivolumab, with fatigue, nausea, and diarrhea being the most common and each reported in 1% of subjects. In comparison, 54% of patients in the docetaxel group experienced mainly neutropenia (27% of cases), febrile neutropenia (10%), leukopenia (8%), fatigue (5%), and anemia (3%). Treatment-related select adverse events of any grade reported in ≥2.5% of patients were rash (9% of patients vs. 3%, respectively, in the nivolumab and docetaxel arm), pruritus (8 vs. 1%), erythema (1 vs. 4%), diarrhea (8 vs. 23%), hypothyroidism (7 vs. 0%), increased alanine aminotransferase levels (3 vs. 1%), increased aspartate aminotransferase (AST) levels (3 vs. 1%), infusion-related reactions (3 vs. 3%), and pneumonitis (3 vs. 0.4%). Grades 3–4 treatment-related select adverse events experienced in patients receiving nivolumab were pneumonitis (1.0% of patients), diarrhea and increased γ-glutamyl transferase levels (each reported in 0.7% of cases) and rash, dermatitis, colitis, increased AST levels, transaminases increased and interstitial lung disease (each reported in 0.3% of patients). Treatment discontinuation due to adverse events occurred in 5% of patients receiving nivolumab (mainly because of pneumonitis) and in 15% of subjects treated with docetaxel (mostly because of fatigue) (23).

#### CheckMate 012: I Line

Recently, the results of the first-line monotherapy with nivolumab for advanced NSCLC in the Phase I, multicohort, CheckMate 012 trial were published. Also in this setting, nivolumab was well tolerated, with 19% of patients reporting Grades 3–4 treatment-related AEs and no treatment-related deaths. According to prior nivolumab data (20–23, 27), treatment-related select AEs affected the skin (any grade, 25%; Grades 3–4, 4%), endocrine (any grade, 14%; Grades 3–4, 0%), gastrointestinal (any grade, 12%; Grades 3–4, 2%), and pulmonary organ (any grade, 6%; Grades 3–4, 2%) (24). These toxicities were easily manageable using established guidelines.

Recently, the results of the cohort of the CheckMate 012 study investigating nivolumab + PT-DC in first-line advanced NSCLC were published. A total of 56 patients were enrolled and treated with the following regimens: nivolumab 10 mg/kg + gemcitabine–cisplatin (squamous) or pemetrexed–cisplatin (non-squamous), or nivolumab 5 or 10 mg/kg + paclitaxel–carboplatin (all histologies). No dose-limiting toxicities occurred during the first 6 weeks of treatment. In patients treated with nivolumab full dose + PT-DC, treatment-related AEs of any grade occurred in 93% of patients, whereas Grade 3/4 AEs occurred in 50% of patients. In the overall population, 95 and 45% of patients experienced any Grade and Grade 3 or 4 treatment-related AEs, respectively. The most frequent (≥30% of patients) treatment-related AEs of any grade were fatigue, nausea, decreased appetite, and alopecia. Regarding treatment-related Grade 3 or 4 AEs, they were mainly (≥5% of patients) pneumonitis, fatigue, and acute renal failure. The majority of patients experienced a treatment-related select AE during the combination period than during nivolumab monotherapy. Treatment-related AEs led to discontinuation of all study therapy in 21% of patients and Grade 3 or 4 treatment-related AEs led to discontinuation in 14% of patients. However, no treatment-related deaths were reported. Because of the high percentage of discontinuation due to AEs, the potential regimen for future indication could be the nivolumab 5 mg/kg + paclitaxel–carboplatin (25).

Recently, the results from CheckMate 026 were presented. The study was one of the first trial in chemotherapy-naïve patients with stage IV or recurrent NSCLC to compare nivolumab with a platinum-based regimen. A total of 541 patients received nivolumab 3 mg/kg every 2 weeks or investigator’s choice of PT-DC every 3 weeks for up to six cycles. Despite an enriched population with PD-L1-positive tumors (threshold defined as ≥1%; *n* = 423), nivolumab did not show superior mPFS compared with chemotherapy (4.2 vs. 5.9 months; HR 1.15, *P* = 0.25) (29).

In this context, the CheckMate 227 Phase III open-label study evaluating platinum-based chemotherapy alone or in combination with nivolumab + ipilimumab or nivolumab in previously untreated advanced NSCLC (NCT02477826) is largely awaited.

#### A Toxicity Profile Never Seen Before

As mentioned, the introduction of immunotherapy in clinical trials showed a specific toxicity profile that is peculiar from the known side effects of cytotoxic chemotherapy or targeted therapies (30). As a result, some patients experienced a novel type of AE considered to be linked to an immune-mediated response directed to different tissues: an immume-related AE (irAE). The percentage of the incidence is around 9%, and the most common irAEs are skin rash, hypothyroidism, diarrhea and colitis, pneumonitis, and increased hepatic function test. These side effects are generally manageable but can be fatal in some cases (31–34). Moreover, their appearance may be subclinical and early diagnosis and management could be extremely challenging. For these reasons, it is important to underline the need to act a careful monitoring of patients receiving nivolumab in order to offer a prompt and optimal management of irAEs. For this reason, physicians should be aware about the use of the established safety guidelines (20, 23, 35, 36). In addition, education of patients and caregivers on recognition of irAEs has a relevant role. Finally, input from other specialties may be valuable for difficult cases (Table 3).

**Table 3:** **Management of selected immune-related adverse events**.

Organ (disorder)	Grade 1–Grade 2	Grade 3–Grade 4
Gastrointestinal (diarrhea colitis)	Supportive care measures	Withheld the drug
	Loperamide	Steroids at 1–2 mg/kg prednisolone or IV equivalent
	If no improvement in 5 days, or if worsening of symptoms, commence steroids at a dose of 0.5–1 mg/kg/day of prednisolone (or IV equivalent)	If no improvement consider infliximab 5 mg/kg
		Grade 4: permanent discontinuation of drug

Dermatologic (diffuse, maculopapular rash)	Manage symptomatically	Grade 3: the drug should be withheld for one dose
	If persistent Grade 2, the drug should be withheld for one dose	Grade 4: permanent discontinuation of drug

Hepatic (elevation in liver function tests)	High-dose IV glucocorticosteroids for 24–48 h, followed by an oral steroid taper (dexamethasone or prednisone)	Grade 3/4: permanent discontinuation of the drug

Lung (pneumonitis)	Observation	Discontinue drug administration
	Delay drug administration	High-dose steroids with methylprednisolone (e.g., 1 g/day IV)
	Consider steroids (e.g., prednisone 1 mg/kg/day PO or methylprednisolone 1 mg/kg/day IV)	Add prophylactic antibiotics
		If not improving after 48 h or worsening, administer additional immunosuppressive therapy (e.g., infliximab, mycophenolate, and immunoglobulins). If improving, taper steroids
		Discontinue treatment permanently

Endocrine (hypophysitis)	Asymptomatic, no intervention needed: monitor only	Withhold the treatment
		Use methylprednisolone 1–2 mg/kg intravenously (IV). This should be followed by prednisone 1–2 mg/kg orally (PO) once daily with gradual tapering over 4 weeks and replacement hormones during the tapering. The drug can be restarted with Grade 2, but Grade 3/4 endocrinopathy requires permanent drug discontinuation

Renal injury	Monitor renal function, promote hydration and cessation of nephrotoxic drugs	Prednisolone 1–2 mg/kg or IV equivalent. Discontinue the drug

Nephritis	Consider prednisolone 0.5–1 mg/kg	

*Adapted from Ref. (35, 36)*.

#### Peculiar Aspects

##### Combination

The combination of nivolumab with different drugs in NSCLC is under investigation. Of note, combinations of the anti-CTLA4 antibody ipilimumab + nivolumab have showed promising results (37), and several trials are ongoing (NCT02477826, NCT02659059, NCT02864251, NCT01454102, and NCT02869789). Toxicity management is a challenging issue, and new dosages and schedules are under evaluation.

##### Onset

The onset of immune adverse events occurs on average 6–12 weeks after starting of therapy. It should be considered that these events can happen within days of the first dose, after several months of treatment, and even after discontinuation of therapy.

#### Open Questions

Currently, many questions are still unsolved. First, the toxicity profile in “real-world,” since patients included in clinical trials do not represent the total population in clinical practice. In this setting, there is a lack of data as well as people with pre-existing autoimmune conditions. In such cases, physicians have to consider if benefit exceeds the risk.

A number of case reports about rare irAEs are publishing in literature demonstrating the need to improve the recognition of clinical abnormalities and their association with nivolumab treatment. The awareness of nivolumab safety will grow as experience of physician will increase as well.

Second, immunotherapy has improved survival and as a consequence, a new set of survivorship issues may arise for management. For instance, there may also be sequelae due to an interplay between late effects of radiotherapy in addition to immunotherapy and association among immunotherapeutics MoAbs or targeted therapies must be deeply explored in order to unveil newer and unexpected safety concerns.

## Nivolumab on Real-World Population: The Strengths and Weaknesses

After the unprecedented clinical results regarding the activity and the long-term response duration even in heavily pretreated NSCLC squamous and non-squamous subtypes, nivolumab quickly became an undebatable gold standard in second-line setting. These results are noteworthy also because adverse events are generally manageable and or reversible.

The strength of nivolumab arose from clinical trials, especially those well-designed Phase III (22, 23). In order to maximize these astonishing results in real-world population, it is necessary to understand in which patients this drug must be employed and in which nivolumab does not work at all. In addition, it is important to highlight the challenging “gray zones” coming from nivolumab experience in the past 2 years of clinical practice.

In squamous and in non-squamous patients, nivolumab shows nearly 20% of RR and approximately two-third of response are durable and persisting with a plateau after more than 24 months of follow-up in overall survival. As a consequence, it has been demonstrated that nivolumab can provide a real control of the disease leading to the concept of disease chronicization. Unfortunately, 80% of patients have a temporary control of the disease, and in the era of precision medicine, it is essential to understand the main reasons. Looking at the cross-over shape of the CheckMate 057 (23) overall survival curves between docetaxel and nivolumab, the main reason for this particular aspect can be due to the activity of the immune checkpoint inhibitor in one undefined subpopulation. This point led investigators to analyze one or more predictive biomarkers, and as a result, PD-L1 tumoral staining has became an important putative biomarker to select the patient who would benefit more with of this class of drugs (38).

Nivolumab has been studied in all-comers patients, regardless of PD-L1 expression; however, a *post hoc* analysis analyzing the percentage of positivity of tumoral PD-L1 was carried out and different cutoff (>1, >5, and >10%) were reported.

In non-squamous histotype, the PD-L1 tumor expression is predictive of nivolumab activity in term of ORR, DOR, mPFS, and mOS. In particular, higher ORRs were observed when PD-L1 was expressed ranging from 31 to 37% respect to 18% in overall population and 9% in PD-L1-negative patients. Median DOR was longer with nivolumab than with docetaxel across different PD-L1 expression levels (16 vs. 5.6 months). Among PD-L1-negative patients responsive to nivolumab, the mDOR was higher respect to docetaxel (18.3 vs. 5.6 months). This result highlights how PD-L1 alone is a defective predictive biomarker.

A further sub-analysis in strong PD-L1-positive tumors (i.e., >50%) has confirmed the axiom “more PD-L1 expression on tumor and more nivolumab clinical activity.” There are many reasons to consider PD-L1 expression as a weak predictive biomarker. First of all, the confounding role between predictivity and prognosis. Many studies associated PD-L1 overexpression with poor prognosis (39); however, prognosis depends on the characteristic of PD-L1 expression and on lymphocyte population forming tumor-infiltrating cells. In fact, CD8 T cells infiltrations strongly correlates with good prognosis in NSCLC, while high B cells and CD4 T cells seem to not impact on prognosis (40–42). It is possible to assume that the subtypes of TILs and the frequency of CD8^+^ T cells infiltrating tumor and PDL1 tumoral expression are all important to predict the activity of nivolumab more than PD-L1 expression alone. In fact, like chronic infection, in cancer antigen, persistency leads to T cell exhaustion with a high number of T reg and other immunosuppressive myeloid cells constituting TILs. In this situation, tumor PD-L1 expression is not enough to predict the activity of nivolumab on the contrary in TILs rich in T cells CD8^+^ even with PD-L1 low expression the immune checkpoint inhibitor could stimulate the awakening of competent immune system.

Some elegant models seem to corroborate this hypothesis: the frequency of CD8^+^ T cells may be associated with better clinical response to immune checkpoint blockade (43, 44), while an immunosuppressive protumoral microenvironment defines intrinsic resistance to anti-PD1 therapy (45). Moreover, myeloid-derived suppressor cells (MDSCs) are recently emerged since they produce many factors stimulating angiogenesis and immunosuppression with a reduction of viability and number of CD8^+^ T cells in TILs (46).

Furthermore, MDSCs accumulate in tumor and blood of NSCLC patients, and they are associated with poor prognosis (47, 48). Their quantity reflects a higher number of neutrophil count and a simple and easy calculation of neutrophil to lymphocyte ratio could be a predictive marker of response to immunotherapy (49, 50).

Regarding clinical features associated with a major probability of response, data from a subgroup analysis showed that smoking habit has an important role, especially in non-squamous histology. Ever smoker has a great possibility to have a clinical benefit from nivolumab as demonstrated from CheckMate 057 study (23). This aspect is related to a higher rate of non-synonimous load mutation due to genetic instability of tumors occurring more in smokers than in never-smokers patients. These neoantigens may elicit an immune response in particular when their expression is represented in most tumor cells generating the theory that a clonal mutation has a better possibility to generate a neoantigen recognized by immune system rather than a subclonal expression (51).

Tumors with low mutational burden seem to benefit less from nivolumab according to a subgroup analysis from CheckMate 057. Moreover, it was shown that EGFR-mutated tumors and never-smokers patients had a similar benefit if treated with docetaxel or nivolumab.

The expression of PD-L1 in tumors harboring EGFR mutations or ALK translocations is generally high; however, no reliable data and final conclusions can be drawn from literature data (52, 53).

Recently, in a larger cohort of EGFR/ALK-positive patients, the lack of expression of PD-L1 and the absence of CD8^+^ T cells in TILs surrounding these tumors were seen. This aspect could classify oncogenic driven tumors as non-inflamed tumors, suggesting a scarce probability to induce an immune awakening and a low activity from immune checkpoint inhibitor agents (54).

The mutational load combined with PD-L1 expression and the analysis of lymphocyte subpopulation of TILs may represent a sort of signature of prediction of response to nivolumab. However, no standard cutoff are available, and there are still many methodological issues regarding the definition of “high” vs. “low” mutational rate tumors.

Nivolumab demonstrates higher efficacy than docetaxel in second line irrespective to PD-L1 expression and in non-squamous patients this benefit increases with the expression of PD-L1. However, the mDOR of nivolumab and its better safety profile renders this drug a reasonable choice even in PDL1-negative patients. This finding led the FDA and EMA approval of nivolumab for all-comers patients and several guidelines do not recommend PDL1 testing.

The issue of a specific predictive biomarker is an important challenge since nivolumab is not a treatment that fits for all patients for several reasons.

First of all the safety: in a *post hoc* analysis from CheckMate 057, a higher risk of death emerged in the first 3 months of treatment with nivolumab respect to docetaxel in particular in poor prognosis patients, especially those with worse ECOG PS and heavy disease burden (55). This aspect is partially explained by a delayed pattern of response of nivolumab, but other characteristics may contribute to contraindicate the use of nivolumab instead of chemotherapy. Second, the sustainability of nivolumab therapy for all patients, in particular in non-squamous histology, across countries.

Some authority regulation agencies like UK National Institute for Health and Care Excellence and Canadian Agency for Drugs and Technologies in Health rejected the use of nivolumab merely due to costs defining this drug as non-cost-effective (56, 57).

Recently, the Swiss Health System conducted a study in order to investigate the cost-efficacy of nivolumab compared with docetaxel. A way to consider this drug effective and sustainable is to select patients with non-squamous histology and testing PD-L1 (cutoff >10%). However, an acceptable ICER threshold of CHF 100,000/QALY is reached only reducing the price of the drug or the dosage or the duration of treatment (58).

It is probable that the absence of a predictive marker of activity will not allow nivolumab to confirm its usefulness largely demonstrated in many trials in a real-world population due to accessibility disparity across countries.

## Overcome the Resistance: Future Strategies

There are two main causes of resistance to immune checkpoint inhibitors: the first one is an intrinsic resistance and the second one is an acquired resistance. The former, excluding the mechanism of pseudo-progression, is due to an immunologic ignorance or an adaptive immune resistance. The combination of PD-L1 expression and TIL presence surrounding and within a tumor may classify carefully this situation (59).

The immune-ignorant phenotype lacks a precise strategy; however, the combination of chemotherapy and nivolumab could switch this situation toward and “immune-awakening” due to the delivery of neoantigens as killing effect to chemotherapy use. In the Phase I multicohort study, CheckMate 012 nivolumab was combined with PT-DC (25). In this non-pretreated cohort, the combination showed a good safety profile and encouraging activity in particular when nivolumab at 5 mg/kg was combined with the paclitaxel–carboplatin regimen leading to a 62% of 2-year OS rate. Data are still immature to definitely suggest the application of this strategy only to immune-adaptive resistance or ignorance. Nevertheless, it is intriguing to think about a different strategy in cases where the use of nivolumab alone predicts a worse clinical benefit.

Another approach is to combine nivolumab with the anti-CTLA4 agent ipilimumab in order to enhance T-cell antitumor activity through distinct and complementary mechanisms.

Based on the sole PD-L1 expression, it could be presumed that in PD-L1-positive tumor nivolumab alone should be enough and in PD-L1-negative tumors the combination with ipilimumab could restore the sensitivity to nivolumab.

Several cohorts of CheckMate 012 explored the combination of different doses of nivolumab and ipilimumab. Recently, the combination of nivolumab 3 mg/kg every 2 weeks + ipilimumab 1 mg/kg every 6 or 12 weeks demonstrated a good tolerability profile and promising efficacy with an ORR of 39–47% with mDOR not reached in first-line treatment (37). Patients with higher levels of PD-L1 expression had especially robust responses to the nivolumab/ipilimumab combination. Among patients with tumor PD-L1 expression levels of ≥50% treated with nivolumab every 2 weeks and ipilimumab Q12w, the ORR was 100% and the median PFS was 13.6 months. However, the nivolumab/ipilimumab combination demonstrated efficacy across all tumor PD-L1 expression levels, even among patients with <1% tumor PD-L1 expression.

The combination of ipilimumab 1 mg/kg q6w + nivolumab 3 mg/kg q2w in PD-L1 unselected population is ongoing in a Phase III trial in first-line treatment (CheckMate 227).

In order to circumvent the intrinsic or acquired resistance, other strategies are under investigation. Early phase trials suggest an activity in particular with the combination with other inhibitors or agonists of immune synapse like Abs targeting CSF1R, LAG3, TIM3, IDO, GITR, and OX40. Finally, the combination of nivolumab and radiotherapy (60) or CAR-engineered T cell ACT and vaccines (61) may represent a fascinating strategy to enhance the activity of nivolumab alone.

In EGFR-positive tumors where there is a lack of response of nivolumab in patients previously treated with TKIs, the research is currently focused on naïve EGFR TKI population. This approach is based on the link between the high probability to generate a response with EGFR TKIs in naïve population and the induction of upregulation of PDL1 and TILs. Nivolumab was studied in pretreated and in EGFR TKIs naïve population with promising results observed in the naïve group (62). With the same rationale nivolumab is currently being studied with crizotinib (NCT01998126) and results are largely awaited.

Another strategy to explore is the combination between immune-checkpoint inhibitors and antiangiogenic agents due to cross talk between this two systems and the possibility to influence the angiogenic power and immune-tolerance against tumor. However, even if the rational is strong, the huge number of factors regulating these two axes renders difficult to forecast the results.

In conclusion, nivolumab currently represents the gold standard for the therapy of advanced, pretreated SCC NSCLC and may represent, with some criticism about the role of PDL tumor expression, a valid option in pretreated nsq NSCLC.

The sustainability and disparity across countries lead the affordability of this drug a main concern for the future. Even if for the first time, we have observed a long and durable response in lung cancers using nivolumab in second line, many questions remain to be answered. In particular, the understanding of the right selection of the patient who would benefit more from the drug and the next step of moving toward a first-line treatment with nivolumab in all-comers to control cancer growth from the beginning.

Finally, it is crucial to understand and overcome the immunoresistance mechanisms in order to develop future studies not only trying a combination based on “*in vitro*” rationale but orienting the discoveries of older trials in biologically based Phase I studies.

Nivolumab is not a “one-size fits all” treatment and the main risk is to deny one of the most powerful drug ever employed in clinical practice.

## Author Contributions

All the authors contributed equally to this paper and agreed to be accountable for the content of the work.

## Conflict of Interest Statement

The authors declare that the research was conducted in the absence of any commercial or financial relationships that could be construed as a potential conflict of interest.
